# Supplementary material for: Charge density wave surface reconstruction in a van der Waals layered material
Source: Nat Commun. 2023 Sep 15;14:5735. doi: 10.1038/s41467-023-41500-6 (PMC10504333; doi:10.1038/s41467-023-41500-6)
Supplement: Supplementary file 1 — Supplementary Information [file 41467_2023_41500_MOESM1_ESM.pdf]

Supplementary Information

Charge density wave surface reconstruction in a van der Waals layered material

Sung-Hoon Lee[1,*] and Doohee Cho[2,†]

[1]*Department of Applied Physics, Kyung Hee University, Yongin 17104, Republic of Korea*

[2]*Department of Physics, Yonsei University, Seoul 03722, Republic of Korea*

Supplementary Note 1. Tight-binding model of a trimer

In a tight-binding framework, the Hamiltonian of a trimer with nearest-neighbor hopping integrals, t_s and t_b, can be expressed as

$$H = \begin{pmatrix} 0 & t_s & 0 \\ t_s & 0 & t_b \\ 0 & t_b & 0 \end{pmatrix}, \tag{1}$$

and its three eigenvectors and eigenvalues are

$$\left| E_\pm = \pm\sqrt{t_s^2 + t_b^2} \right\rangle = \begin{pmatrix} t_s \\ \mp\sqrt{t_s^2 + t_b^2} \\ t_b \end{pmatrix}, \qquad |E_0 = 0\rangle = \begin{pmatrix} t_b \\ 0 \\ -t_s \end{pmatrix}. \tag{2}$$

The solutions for three special cases are

- $t_s/t_b \approx 1$ (corresponding to the *A*-interface single-layer (trilayer) surface)

$$\left| E_\pm = \pm\sqrt{2}t_b \right\rangle = \begin{pmatrix} 1 \\ \mp\sqrt{2} \\ 1 \end{pmatrix}, \qquad |E_0 = 0\rangle = \begin{pmatrix} 1 \\ 0 \\ -1 \end{pmatrix}. \tag{3}$$

- $t_s/t_b \approx 3/5$ (corresponding to the *M*-interface single-layer surface)

$$\left| E_\pm = \pm\frac{\sqrt{34}}{5}t_b = \pm 1.17t_b \right\rangle = \begin{pmatrix} 3/5 \\ \mp\sqrt{34}/5 \\ 1 \end{pmatrix}, \qquad |E_0 = 0\rangle = \begin{pmatrix} 1 \\ 0 \\ -3/5 \end{pmatrix}. \tag{4}$$

- $t_s/t_b \approx 1/3$ (corresponding to the *L*-interface single-layer surface)

$$\left| E_\pm = \pm\frac{\sqrt{10}}{3}t_b = \pm 1.05t_b \right\rangle = \begin{pmatrix} 1/3 \\ \mp\sqrt{10}/3 \\ 1 \end{pmatrix}, \qquad |E_0 = 0\rangle = \begin{pmatrix} 1 \\ 0 \\ -1/3 \end{pmatrix}. \tag{5}$$

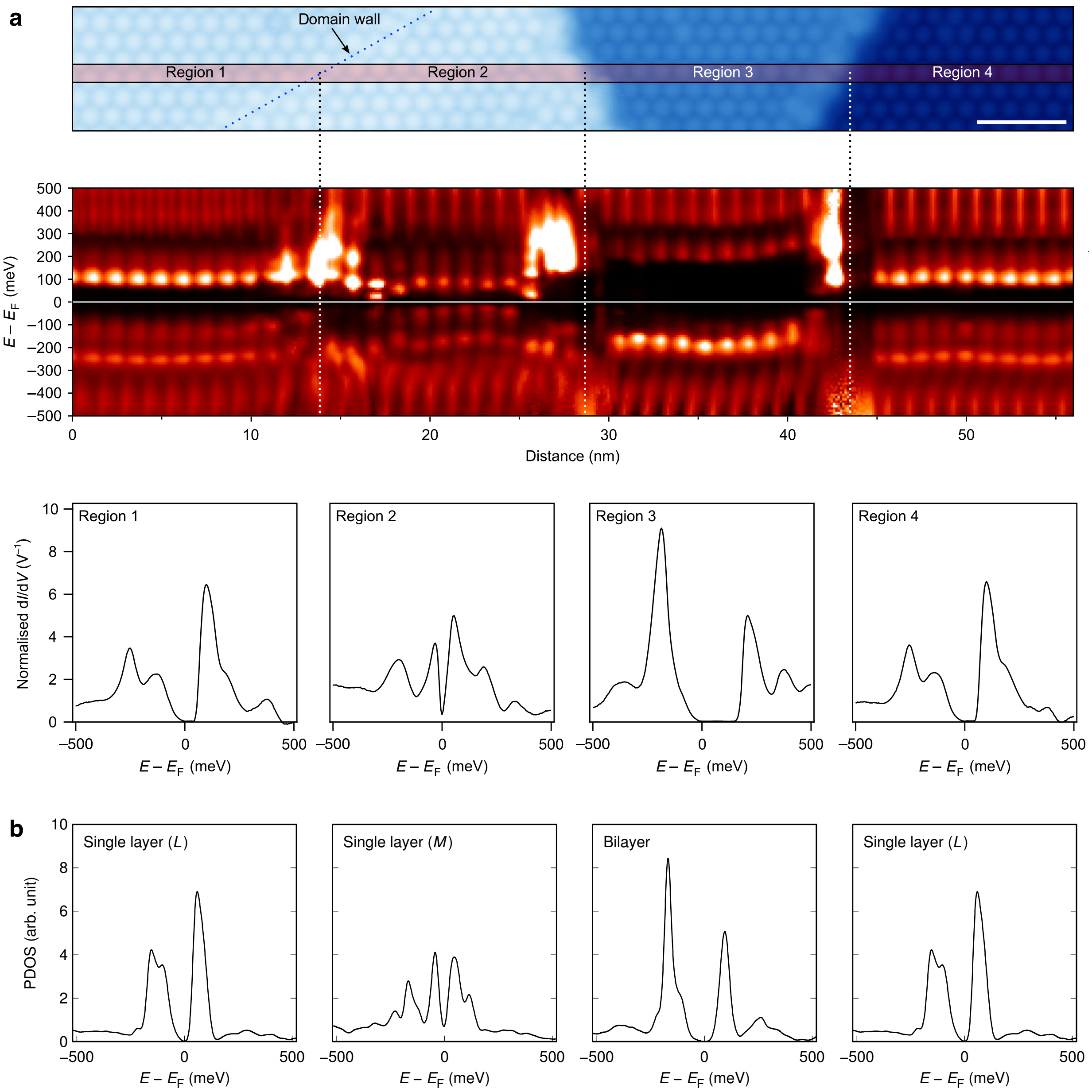

Supplementary Fig. 1. Comparison of **a** the STM dI/dV data (from *Nat. Commun*. 11, 2477 (2020)) and **b** the corresponding PDOS data of this work. It was indistinguishable whether the stacking interface of Region 2 was *M* or *B*. Here, we compare Region 2 with the *M*-interface single-layer surface.

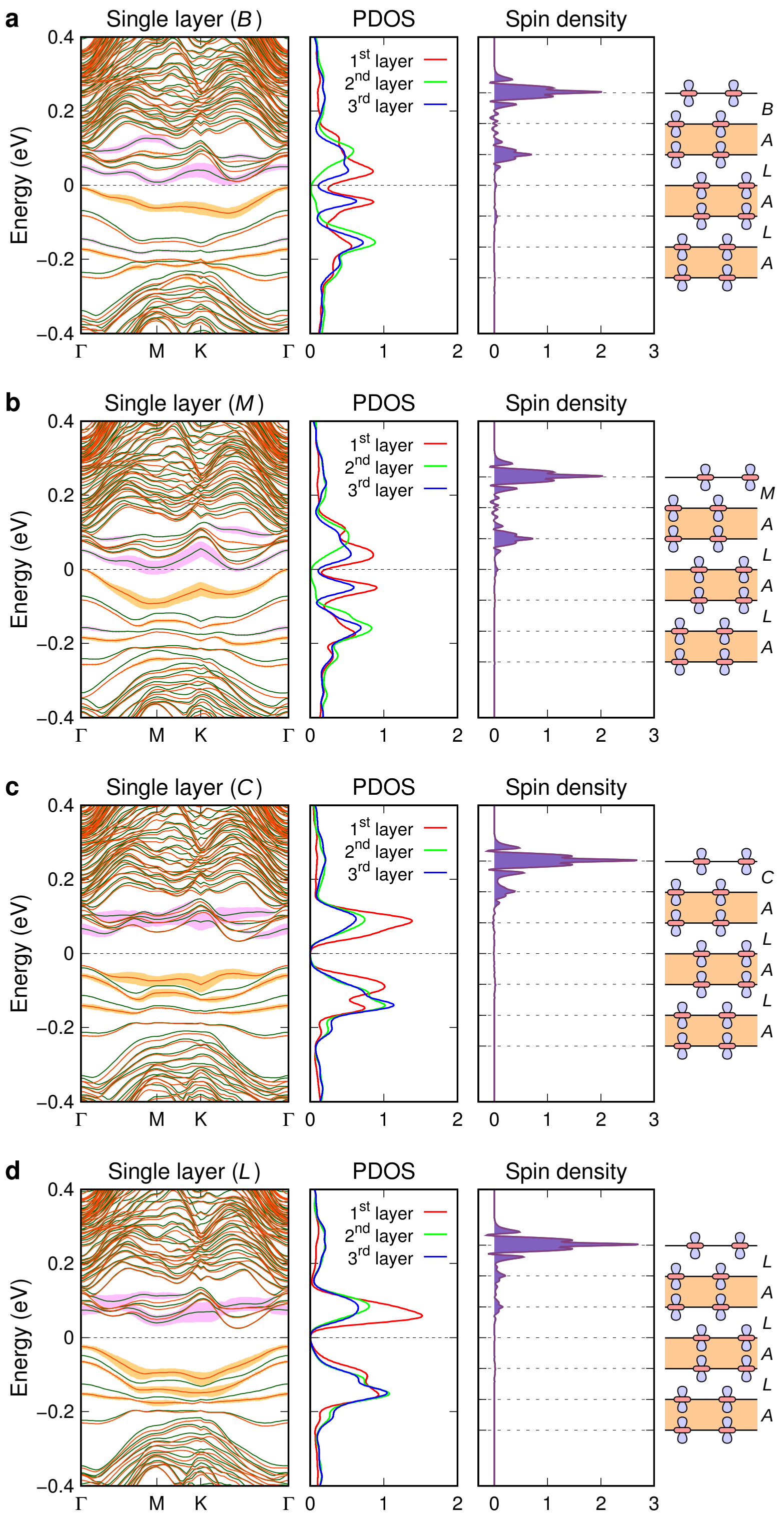

Supplementary Fig. 2. Band structure, PDOS, and spin density of the single-layer-terminated surface for four stacking interfaces: **a** *B*, **b** *M*, **c** *C*, and **d** *L*. In the band structures, the majority and minority spin bands are depicted by red and green lines, respectively. The shaded area (orange and violet) associated with each band represents the spectral weight of the central Ta $5d_{z^2}$ orbital in the top layer.

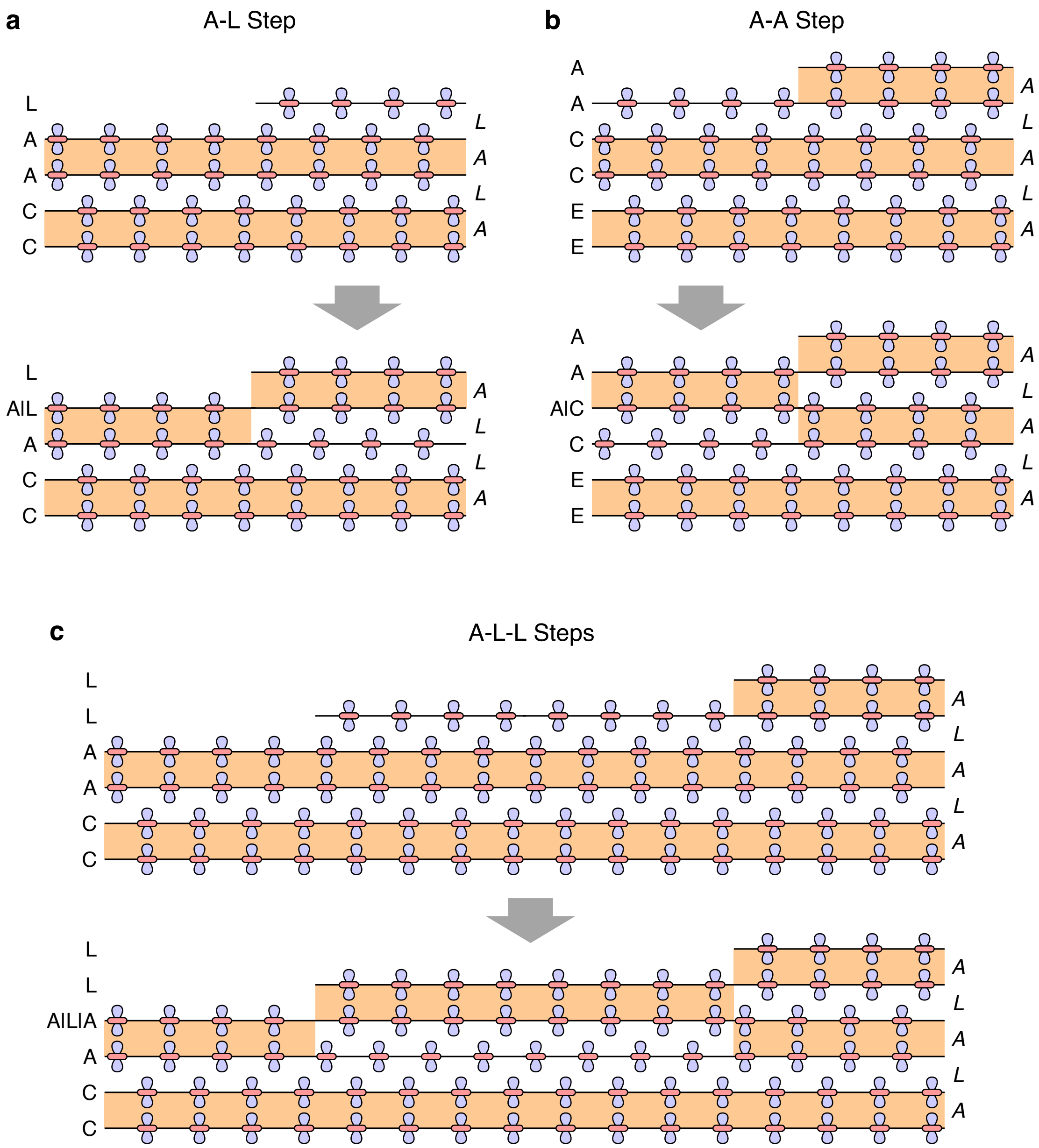

Supplementary Fig. 3. CDW surface reconstruction at single-layer steps. **a** A-L type single-layer step, **b** A-A type single-layer step, and **c** two consecutive steps consisting of A-L and A-A type single-layer steps. This process involves a CDW shift in the second layer without any modifications to the other layers.
